# After the RCT: who comes to a family-based intervention for childhood overweight or obesity when it is implemented at scale in the community?

**DOI:** 10.1136/jech-2014-204155

**Published:** 2014-10-07

**Authors:** James Fagg, Tim J Cole, Steven Cummins, Harvey Goldstein, Stephen Morris, Duncan Radley, Paul Sacher, Catherine Law

**Affiliations:** 1 Centre for Paediatric Epidemiology and Biostatistics, UCL Institute of Child Health, London, UK; 2 Department of Social and Environmental Health Research, London School of Hygiene and Tropical Medicine, London, UK; 3 Centre for Multilevel Modelling, University of Bristol, Bristol, UK; 4 Department of Applied Health Research, UCL, London, UK; 5 Carnegie Faculty, Leeds Metropolitan University, Leeds, UK; 6 Childhood Nutrition Research Centre, UCL Institute of Child Health, London, UK

**Keywords:** OBESITY, INEQUALITIES, CHILD HEALTH, Outcome Research Evaluation, HEALTH BEHAVIOUR

## Abstract

**Background:**

When implemented at scale, the impact on health and health inequalities of public health interventions depends on who receives them in addition to intervention effectiveness.

**Methods:**

The MEND 7–13 (Mind, Exercise, Nutrition…Do it!) programme is a family-based weight management intervention for childhood overweight and obesity implemented at scale in the community. We compare the characteristics of children referred to the MEND programme (N=18 289 referred to 1940 programmes) with those of the population eligible for the intervention, and assess what predicts completion of the intervention.

**Results:**

Compared to the MEND-eligible population, proportionally more children who started MEND were: obese rather than overweight excluding obese; girls; Asian; from families with a lone parent; living in less favourable socioeconomic circumstances; and living in urban rather than rural or suburban areas. Having started the programme, children were relatively less likely to complete it if they: reported ‘abnormal’ compared to ‘normal’ levels of psychological distress; were boys; were from lone parent families; lived in less favourable socioeconomic circumstances; and had participated in a relatively large MEND programme group; or where managers had run more programmes.

**Conclusions:**

The provision and/or uptake of MEND did not appear to compromise and, if anything, promoted participation of those from disadvantaged circumstances and ethnic minority groups. However, this tendency was diminished because programme completion was less likely for those living in less favourable socioeconomic circumstances. Further research should explore how completion rates of this intervention could be improved for particular groups.

## Introduction

Overweight and obesity has been described as the primary childhood health problem in developed nations.^1^ In 2011, estimates from UK data showed that a third of children aged between 2 and 15 were overweight or obese, with prevalence among those living in the most deprived circumstances double that of those living in the least deprived.^2^ While prevalence may be plateauing,^2^ ^3^ the costs for children and families, health services and society remain substantial.^4^

Research on weight management programmes for children who are already overweight or obese has tended to focus on the effectiveness of interventions under research conditions. These studies suggest that family-based interventions may result in moderate reductions in body mass index (BMI).^5^ However the population impact on health and health inequalities of such interventions, when implemented at scale, depends not only on how effective the intervention itself is, but also on who receives it.^6^ ^7^ For example, families living in more favourable socioeconomic circumstances may be more likely to access and adhere to services which support behaviour change.^8^ ^9^ To understand whether implementation is successful, evidence is needed about who is referred to, who starts and who completes potentially effective interventions delivered at scale in the community in relation to the population in need.^10^ ^11^

Recent work mapping weight management interventions for overweight or obese children^12^ identified approximately 50 schemes operational around 2008 in England. In this paper we examine the largest of these schemes, MEND 7–13 (MEND—Mind, Exercise, Nutrition Do It!) in order to achieve two objectives: first, to explore whether the sociodemographic characteristics of MEND participants match those of the population eligible for the intervention; and second, to estimate what characteristics of children, their families and neighbourhoods are associated with completion of a MEND programme. Further objectives^13^ on the differential outcomes^14^ and economic costs of attending MEND, and its salience and acceptability to families and commissioners^15^ are reported elsewhere.

## Methods

### MEND intervention

MEND is a multicomponent family-based community intervention which aims to support families of overweight or obese children (hereafter referred to as overweight, defined as exceeding the 91st centile of BMI (weight/height^2^) of the UK 1990 growth charts^16^) to adopt and sustain healthier lifestyles. The intervention was demonstrated to be effective in reducing BMI of obese children after 6 and 12 months in a randomised controlled trial.^17^

The intervention addresses diet and physical activity through education, skills training and motivational enhancement. Owing to the importance of family involvement for behaviour change, the intervention requires a parent or carer to attend all 20 sessions and the MEND intervention was developed to be delivered in community settings such as schools or leisure centres.^17^ Children are eligible if they are aged between 7 and 13 years old and overweight although those aged 6 (N=298) when they were referred were included in the analyses.

MEND had two operating arms in the UK: MEND Central, a limited company, and MEND Places, a not-for-profit company originally set up to provide a route for donations to subsidise places for families on the MEND programme. When the data for our study were collected, MEND Central described itself as a social enterprise—an ethical business which aims to benefit society in general. All those commissioning MEND between 2007 and 2010 placed funding through MEND Central. Families did not pay to attend the programme, places were funded by a variety of organisations including: the UK Big Lottery Fund (n=8972); NHS Primary Care Trusts (n=4373); non-governmental organisations (n=617); and other non-profit making organisations and a private company (n=36).

**Figure 1:**
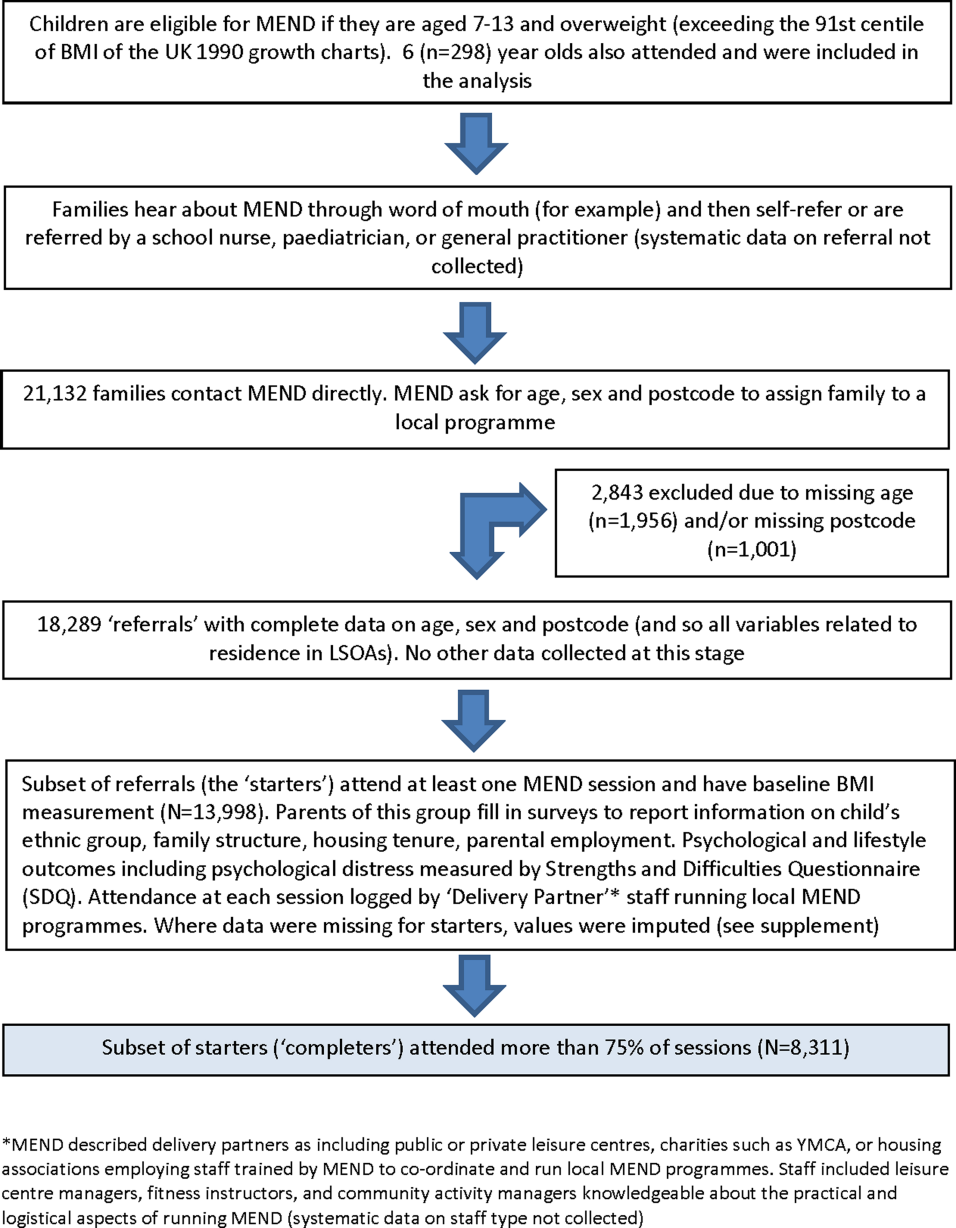
Derivation of samples of MEND participants for analysis.

Between 2007 and 2010, the MEND intervention was implemented on a large scale, with programmes rolled out across all regions of England (see figure 1 for more details). Families referred to the programme contacted MEND (‘referrals’)—whereupon MEND logged the child's age and sex, and the family postcode, collecting no other data. Families who went on to attend one or more sessions (‘starters’) also had sociodemographic and other data collected.

The intervention was delivered in local programmes by ‘delivery partner’ organisations. Intervention content and training was provided to delivery partners by MEND Central. The height, weight and psychological distress of participants were measured at the first and penultimate sessions of each MEND programme. Delivery partners were trained to measure height and weight (which was used to calculate BMI) and also recorded individual attendance of each participant at each session. Psychological distress was reported by parents using the Strengths and Difficulties Questionnaire (SDQ).^18^ Parents also reported the participant's ethnicity (White, Asian, Black and Other) and family socioeconomic circumstances including: family structure (lone parent/carer, couple parents/carers); housing tenure (owner occupied, social rented, private rented); and employment status of the ‘primary earner’ (employed, unemployed). Delivery partners recorded data in an online database collated by MEND Central. For this study, a copy of this database for the period January 2007 to December 2010 was transferred to University College London (UCL) Institute of Child Health for analysis.

The full unit postcode of each MEND participant's residential address was used to assign each address to its respective Lower Super Output Area (LSOA). Each LSOA was then linked to a measure of neighbourhood deprivation (deciles of the Income Deprivation Affecting Children Index (IDACI) 2007).^19^ IDACI measures the percentage of income-deprived households (those in receipt of means-tested benefits such as jobseekers allowance or child tax credits) with children aged 0–15 in the LSOA. Deciles of the index were calculated relative to the deprivation ranking of all English LSOAs; participants in decile 10 live in the 10% most deprived areas in England. We also linked a national urban/rural status (urban, suburban, rural) classification^20^ to each LSOA. MEND data were used to derive how many children attended each MEND programme (hereafter referred to as ‘programme group size’) and the number of programmes which a local programme manager had managed.

In the absence of an agreed definition of completion for family-based interventions for childhood overweight,^21^ children were considered to have completed the programme (‘completers’) if they had attended more than 75% (more than 15 out of 20) of sessions.

Data were complete for BMI, postcode (and so all variables related to residence in LSOAs) and programme level variables. Starters and completers were missing data for the following variables (percentages reported for starters): baseline SDQ (7%), ethnicity (33%), family structure (36%), housing tenure (35%), employment status (63%) and percentage of sessions attended (42%). We followed the guidelines of Sterne *et al*^22^ for the analysis and reporting of missing data. Full details are given in the supplement and summarised here. First, those with complete and incomplete data were statistically different, although differences were small. Second, missingness varied between MEND programmes. Third, excluding all those with any missing data from the MEND data would have reduced the sample (and therefore the statistical power to estimate proportions) from 13 998 to 2787. To minimise bias, adjust for multilevel missingness, and maximise power, we used a multilevel multiple imputation model. The model assumed that data were missing at random; the rationale for this is described in the supplement along with the variables included in the model and the reasons for their inclusion. Ten imputed data sets were produced, analysed separately and results combined using Rubin's rules.^23^ To test whether our findings were influenced by using imputed data we also conducted sensitivity analyses, including analysis using complete case data with and without the variable describing parental employment status, where missingness was particularly high.

### Statistical analysis

We described the population eligible for MEND (‘the MEND-eligible population’) using the Health Survey for England (HSE), an annual, nationally representative cross-sectional survey of English children and adults.^24^ We did this by pooling the data from the 2007 to 2010 surveys for 6–13-year-olds (n=13 468) who were overweight (n=2799, after exclusion of those where valid BMI data was missing (n=1577). All analyses accounted for the HSE complex survey design and sample weights.

The sociodemographic characteristics of children participating in MEND were compared with those in the MEND-eligible population. Differences in percentages, and their statistical significance levels were calculated (MEND-eligible subtracted from MEND). The social gradient of residence by IDACI was compared graphically and tested using linear regression with an interaction term included to test the statistical significance of differences between slopes.

We estimated multilevel (participants clustered within programmes) Poisson regression models^25^ to assess whether completion was associated with baseline (ie, at the first session) age, sex, ethnicity, psychological distress, BMI, family structure, housing tenure, parental employment status, IDACI and urban/rural status. Unadjusted relative risks (uRR) of completion were first estimated in a set of single variable models. Where uRRs were significant at the 5% level, these variables were retained in a multivariable model to allow estimation of adjusted relative risks (aRR).

The following software was used: REALCOM-IMPUTE^26^ (multilevel multiple imputation model), MLwiN^27^ and runmlwin^28^ (multilevel Poisson regression models), and Stata 12.1 (all other analyses).

Parents gave informed written consent for their child to participate in the MEND programme and for their data to be used after anonymisation. Approval for the transfer of the anonymised data from MEND to researchers at the UCL Institute of Child Health, and for the secondary analysis of these data was granted by the UCL ethics committee in October 2010 (ref 2677/002).

## Results

Between January 2007 and December 2010, 21 132 referrals contacted MEND, 18 289 of whom had complete data (see figure 1). A total of 13 998 of these were starters (attended at least one session) and of these 8311 were completers (attended more than 75% of sessions).

**Figure 2:**
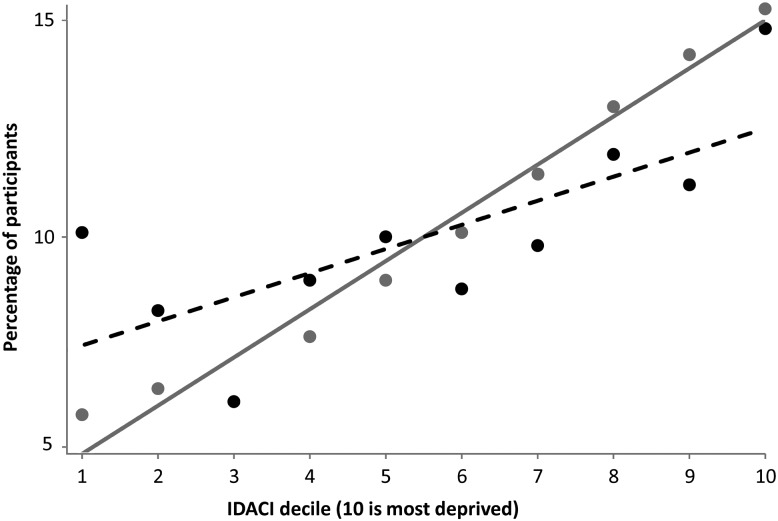
Social gradients of those referred to Mind, Exercise, Nutrition Do It! (MEND; grey dots and line) and MEND-eligible population (black dots and dashed line) by IDACI 2007 (neighbourhood deprivation) deciles. Trends were tested for differences (p<0.001). IDACI, Income Deprivation Affecting Children Index.

Compared to the MEND-eligible population, proportionally more MEND referrals were girls and from urban areas while fewer were from suburban or rural areas (table 1). As would be expected, given the social gradient in childhood overweight, proportionally more of both MEND-eligible children and MEND referrals lived in more deprived compared to less deprived areas but this social gradient was significantly more pronounced for MEND referrals (test for interaction p<0.01: figure 2).

**Table 1:** Sociodemographic differences between the MEND-eligible population and those referred to, who start and who complete MEND

Variables	MEND-eligible population (N=2799) %	MEND participants								
		Referrals (N=18 289)			Starters (N=13 998)*			Completers (N=8311)*		
		Per cent	Diff.	(p Values)	Per cent	Diff.	(p Values)	Per cent	Diff.	(p Values)
Adiposity										
Overweight excl. obese	53.8	NA	NA	NA	15.7	−38.1	(<0.001)	15.8	−38.0	(<0.001)
Obese	46.2	NA	NA	NA	84.3	+38.1	(<0.001)	84.2	+38.0	(<0.001)
Sex										
Boy	53.0	46.7	−6.3	(<0.001)	45.9	−7.1	(<0.001)	43.4	−9.6	(<0.001)
Girl	47.0	53.3	+6.3	(<0.001)	54.1	+7.1	(<0.001)	56.6	+9.6	(<0.001)
Ethnicity										
White	79.6	NA	NA	NA	77.3	−2.3	(0.01)	78.5	−1.1	(0.3)
Asian	10.0	NA	NA	NA	13.0	+3.0	(<0.001)	12.4	+2.4	(<0.003)
Black	5.7	NA	NA	NA	5.9	+0.2	(0.6)	5.5	−0.2	(0.6)
Other	4.7	NA	NA	NA	3.8	−0.9	(<0.03)	3.6	−1.1	(0.02)
Family structure										
Lone parent	30.5	NA	NA	NA	34.5	+4.0	(0.02)	31.5	+1.0	(0.6)
Couple	69.5	NA	NA	NA	65.5	−4.0	(0.02)	68.5	−1.0	(0.6)
Housing tenure										
Owned	63.5	NA	NA	NA	53.4	−10.1	(<0.001)	58.2	−5.3	(<0.001)
Social	25.2	NA	NA	NA	31.9	+6.7	(<0.001)	27.9	+2.7	(0.009)
Private	11.3	NA	NA	NA	14.7	+3.4	(<0.001)	13.9	+2.6	(0.001)
Employment status										
Employed	79.4	NA	NA	NA	73.9	−5.5	(<0.001)	77.2	−2.2	(0.04)
Unemployed	20.6	NA	NA	NA	26.1	+5.5	(<0.001)	22.8	+2.2	(0.04)
Urban/rural										
Urban	82.1	89.2	+7.1	(<0.001)	89.0	+6.9	(<0.001)	88.1	+6.0	(<0.001)
Suburban	8.9	6.4	−2.5	(<0.001)	6.4	−2.5	(<0.001)	7.0	−1.9	(0.001)
Rural	9.0	4.6	−4.4	(<0.001)	4.5	−4.5	(<0.001)	4.9	−4.1	(<0.001)

*Proportions for MEND ethnicity, family structure, housing tenure and employment status of ‘primary earner’ for starters and completers were based on data where missing values were imputed. All other proportions based on complete data.

‘Diff.’, difference between percentages of MEND-eligible population and MEND participants (MEND percentage—MEND-eligible percentage). ‘NA’, BMI, Ethnicity and family socioeconomic variables collected at the first session of the programme and so not available at referral.

BMI, body mass index; MEND, Mind, Exercise, Nutrition, Do It!.

Compared to the MEND-eligible population, proportionally more MEND starters were: obese rather than overweight but not obese; girls; Asian; from families with a lone parent; living in social or private rented accommodation; living in a family where the primary earner was unemployed; or resident in urban areas (table 1). Proportionally fewer were children whose ethnicity was reported to be White or ‘Other’. Similar sociodemographic patterns were seen in those who completed a MEND programme (table 1). Comparisons of both MEND starters and completers with the MEND-eligible population by neighbourhood deprivation deciles showed similar differences in slopes to the differences for MEND referrals (data not shown).

For sensitivity, the differences in percentages between the MEND-eligible population and MEND starters and completers were also calculated: excluding children who were 6 years old; and separately for those MEND children who were obese and for those who were overweight but not obese. Results were similar to those reported in table 1 for all MEND participants (data not shown).

Single (unadjusted) variable models showed that, having started a MEND programme, completion was significantly associated with psychological distress at baseline, sex, family structure, housing tenure, employment status, IDACI 2007, programme group size and the number of programmes per manager (see uRR in table 2). These variables were retained in the multivariable model. Age, baseline BMI, ethnicity and urban/rural status were not significantly associated and therefore not retained.

**Table 2:** Unadjusted (uRR) and adjusted relative risks (aRR) of completion of a MEND programme by sociodemographic characteristics of participants (N=13 998)

Parameters	Single variable models	Multivariable model
	uRR (95% CI)	aRR (95% CI)
Intercept	–	0.85 (0.76 to 0.95)**
BMI baseline (ref. 91st–95th centile)		
95th–98th centile	1.02 (0.91 to 1.14)	–
Greater than 98th centile	1.00 (0.92 to 1.10)	–
SDQ baseline (ref. ‘Normal’)		
‘Borderline’	0.95 (0.89 to 1.02)	0.97 (0.90 to 1.04)
‘Abnormal’	0.88 (0.83 to 0.93)***	0.91 (0.86 to 0.97)**
Age (years)	0.99 (0.97 to 1.00)	
Sex (ref. Girls)		
Boys	0.90 (0.86 to 0.95)***	0.91 (0.87 to 0.96)***
Ethnicity (ref. White)		
Asian	0.94 (0.87 to 1.00)	–
Black	0.91 (0.82 to 1.00)	–
Other	0.94 (0.83 to 1.08)	–
Family structure (ref. couple)		
Lone parent	0.87 (0.82 to 0.92)***	0.93 (0.88 to 0.98)*
Housing tenure (ref. Owner occupied)		
Social rented	0.80 (0.75 to 0.85)***	0.88 (0.82 to 0.95)***
Private rented	0.86 (0.80 to 0.92)***	0.90 (0.84 to 0.97)**
Employment status (ref. Employed)		
Unemployed	0.84 (0.79 to 0.89)***	0.93 (0.87 to 0.98)*
IDACI 2007 deciles (ref. Decile 1, least deprived)		
2	1.00 (0.88 to 1.14)	1.01 (0.89 to 1.15)
3	0.97 (0.86 to 1.09)	0.99 (0.88 to 1.12)
4	0.95 (0.84 to 1.08)	0.98 (0.87 to 1.11)
5	0.92 (0.82 to 1.04)	0.96 (0.84 to 1.08)
6	0.87 (0.78 to 0.97)*	0.92 (0.82 to 1.03)
7	0.86 (0.77 to 0.97)*	0.93 (0.82 to 1.04)
8	0.84 (0.75 to 0.95)**	0.93 (0.82 to 1.05)
9	0.81 (0.73 to 0.91)***	0.91 (0.80 to 1.02)
10—most deprived	0.74 (0.66 to 0.83)***	0.85 (0.76 to 0.96)**
Urban/rural status (ref. Urban)		
Suburban	1.09 (1.00 to 1.20)	–
Rural	1.10 (0.98 to 1.22)	–
Programme group size (ref. 1–5)		
6–9	0.93 (0.86 to 1.01)	0.93 (0.86 to 1.01)
10 or more	0.83 (0.76 to 0.90)***	0.84 (0.77 to 0.91)***
Number of programmes per manager (ref. less than 10)		
10 or more	0.91 (0.85 to 0.98)*	0.93 (0.86 to 0.99)*

*p<0.0.5, **p<0.01, ***p<0.001.

BMI, body mass index; IDACI, Income Deprivation Affecting Children Index; SDQ, Strengths and Difficulties Questionnaire.

In the multivariable model, the aRR (see aRR in table 2) showed that children who had started a MEND programme were significantly less likely to complete it if they: were reported by parents as having ‘abnormal’ rather than ‘normal’ levels of psychological distress; were boys; were from lone parent rather than couple parent families; lived in social or private rented rather than owner occupied accommodation; lived in a family where the primary earner was unemployed rather than employed; were resident in LSOAs in more deprived IDACI 2007 deciles rather than less deprived; participated in a relatively large MEND programme group; or participated in a programme led by a manager who had run a relatively high number of programmes.

In the absence of an agreed definition of completion we re-estimated the multivariable model with completion defined as attending more than 60% of sessions. Findings were similar to those observed for completion defined as attendance at more than 75% of sessions (data not shown).

We repeated all analyses with complete case data to test sensitivity of findings to imputation and specifically to the high proportion of missing data on employment status. Findings were similar to those using imputed data (see online supplementary section G12).

## Discussion

Our results showed that in comparison to the MEND-eligible population, proportionally more children who started or completed MEND lived in less favourable socioeconomic circumstances (indicated by employment of primary earner, family structure and housing tenure). Relative to the MEND-eligible population, proportionally more of those who started or completed a MEND programme were girls, Asian and urban dwelling and proportionally fewer were of White or ‘Other’ ethnicity. Finally, relative to the MEND-eligible population, proportionally more MEND participants were obese rather than overweight but not obese. These differences may have arisen from differential uptake or differential provision of MEND but we did not have data to investigate this further.

In contrast, for those who started a MEND programme, those living in less favourable socioeconomic circumstances were less likely to complete it, as were boys and those reporting ‘abnormal’ levels of psychological distress at baseline. Completion of a MEND programme was also less likely if the programme group size was relatively high and if the programme manager was more experienced. Completion was not associated with baseline BMI, age or ethnic group. However, these relative differences in completion rates were not sufficient to remove the tendency for starters and completers overall to be less advantaged than the MEND-eligible population.

## Strengths and limitations

We used data from a family-based intervention for childhood overweight which had been implemented at scale in the community. This was an unusual opportunity to assess how an intervention which has been found to be effective in a research setting is provided and accessed in the ‘real world’. In addition, the large scale implementation meant that the data set was sufficiently large to precisely estimate proportions for each sociodemographic group and factors associated with predictors of completion of the programme. The MEND data were collected for service rather than research purposes and, as is common in service data, data quality was variable, with considerable missing data. We carried out extensive data cleaning procedures to maximise the quality of the data that we analysed. We also used imputed data obtained from a multilevel multiple imputation model to mitigate systematic bias and increase the precision of estimates. We also ran analyses with both complete case and imputed analyses to show that the overall conclusions we drew were the same.

We used a robust, nationally representative survey (HSE) to estimate the population of children in England who would theoretically be eligible for the MEND intervention, and compare the characteristics of this population ‘in need’ with those who were referred to, started or completed a MEND programme. The lone parent and employment status variables were not coded identically in MEND and the HSE. However, our results using the lone parent and employment variables were consistent with those using the IDACI 2007 measure which was standard in both MEND and HSE data sets.

We repeated our HSE-MEND comparison, substituting two other contemporaneous nationally representative data sets in turn in the place of the HSE. The Millennium Cohort Study sweep four^29^ was nationally representative for overweight children in England aged 6–8 years old in 2008 and, when compared to MEND participants of the same age, findings were similar to the HSE-MEND comparison (eg, proportionally more children living in less favourable circumstances among the MEND participants than in the Millennium Cohort). The National Child Measurement Programme^30^ was nationally representative for overweight children in England in school year 10 (10–11 years old) in the years 2007–2010 and, when compared to MEND children of the same age, findings were again similar to the HSE-MEND comparison. These sensitivity analyses (data not shown) showed that our findings were robust to how we assessed the population who would be eligible for MEND.

## Comparison with other studies

Differential provision or uptake of interventions both have the potential to generate, maintain, widen or decrease health inequalities.^7^ ^8^ We found no research which compared the sociodemographic characteristics of participants in a family-based community intervention for childhood overweight with the population in need, although a similar analysis has been outlined in an evaluation framework for an Australian weight management scheme for adults.^31^ Our findings that the sociodemographic characteristics of MEND participants marginally over-represent families living in more deprived areas are consistent with previous work on smoking cessation^6^ and coronary heart disease treatment.^32^ However, we are not aware of similar work in services for children's health.

Relatively few published studies have considered who ‘drops out’ or completes children's weight management interventions, and a review of this work suggests that study designs (including intervention type) and findings are heterogeneous and typically based in research or clinical settings.^33^ This study adds to this evidence base because it was based on a large data set who received a family-based community intervention for childhood overweight following implementation at scale in the community (as opposed to in a research setting).

Consistent with smaller scale studies of attrition in paediatric weight management interventions delivered predominantly in clinical settings, we found that completion was less likely among participants living in less favourable socioeconomic circumstances.^9^ ^34^ ^35^ No families had to pay for the MEND programme and so direct financial costs of participation would not explain differential completion by socioeconomic status. However, indirect costs of participation to families and the amount of time available to parents to take children to programmes may have explained some of these differences.^33^ This reinforces concerns that the acceptability of, or adherence to, services is worse for those from less advantaged circumstances and may indicate that services still need to develop and adapt to meet the needs of these families.

## Conclusions

The provision and/or uptake of MEND did not appear to compromise and, if anything, promoted participation of those from more disadvantaged circumstances and from ethnic minority groups. This suggests that participation in MEND, when it was implemented at scale across England, had the potential to make a contribution to tackling health inequalities. However, this potential was diminished to some extent because; having started a programme, completion was relatively less likely for those participants living in less favourable socioeconomic circumstances. It was also less likely for boys. Further research should explore why this occurs, and investigate how completion rates could be improved for these groups. Approaches to this may include using survival analysis to examine critical points where exit from the programme is more likely.

MEND programmes have been implemented across a number of income rich countries beyond England, including Wales, Denmark, Australia, New Zealand, USA and Canada and so the results from this study may be generalisable to these countries. We do not know to what extent our findings are generalisable to other weight management or community interventions. Assessment of the reach of interventions (of any kind) when implemented at scale is likely to be based on service or routine data, and such data are likely to have higher levels of missingness than research data. We hope that the publication of these findings will stimulate further exploration of such data to determine what happens when research is put into practice in community settings.

**What is already known on this subject**

- Differential referral to, participation in and completion of interventions have the potential to generate, maintain, decrease or widen inequalities in health.
- Childhood overweight and obesity are already socially patterned so the potential for interventions to widen inequalities is of particular concern.
- The sociodemographic characteristics of participants in weight management randomised controlled trials may not reflect those of the population who would be eligible for the intervention if it was implemented at scale.

**What this study adds**

- Compared to those eligible for the intervention, proportionally more girls, children who lived in less favourable socioeconomic circumstances and Asian children started and completed Mind, Exercise, Nutrition Do It! (MEND).
- In contrast, having started a MEND programme, children were relatively less likely to complete it if they lived in less favourable socioeconomic circumstances and were boys. There were no differences in completion by ethnic group.
- Participation in MEND, when it was implemented at scale across England, had the potential to make a contribution to tackling health inequalities. However, this potential was diminished to some extent because, having started a programme, completion was relatively less likely for participants living in less favourable socioeconomic circumstances.

## Supplementary Material

Web supplement
