# Supplementary material for: After the RCT: who comes to a family-based intervention for childhood overweight or obesity when it is implemented at scale in the community?
Source: J Epidemiol Community Health. 2014 Oct 7;69(2):142–8. doi: 10.1136/jech-2014-204155 (PMC4316870; doi:10.1136/jech-2014-204155)
Supplement: Web supplement [file jech-2014-204155-s1.pdf]

Supplement to Fagg et al submission to JECH 2014: missingness and multiple imputation

S1. Introduction

This supplement refers to an analysis of the differences in socio-economic characteristics of children attending a weight management intervention delivered under service conditions, described in a paper submitted to the Journal of Epidemiology and Community Health (*"After the RCT - who comes to a family-based intervention for childhood overweight or obesity when it is implemented at scale in the community?"*). This supplement assumes that the reader has read the article to which this document refers and therefore does not define terminology or explain analyses which are described in that article. Data analysed were collected by the staff delivering the intervention. In line with other service level and routine data sources, the data were subject to relatively extensive missingness. This supplement briefly describes: key background assumptions about the nature of missing data for the general reader; the extent, patterns and inter-relationships of missing observations in the dataset; the approach taken to mitigate potential sources of bias including a consideration of an alternative approach; and data from sensitivity analyses referred to in the paper.

The supplement refers to analyses of MEND starters and completers only (analyses of those referred to MEND were based on completely observed data).

S2. Background and theory

Missing data is an issue in most research settings and arise when researchers (or services in this case) intend to collect information but do not. Three broad processes, described by Rubin,[1] can lead to missing data: missing completely at random (MCAR), missing at random (MAR), and missing not at random (MNAR). All methods using data with missing values make an assumption that the missing data were introduced into their datasets by one or more of these processes. These assumptions are made because there are few applied situations where the reasons for data to be missing can be determined.

Complete case analysis, a common analytical approach, excludes individuals who have missing data on any variable (incomplete data) from the analysis. This approach assumes that complete cases are a random subsample; that missing values were introduced into the analysis completely at random (MCAR). This approach can be adequate in situations where differences between those individuals with complete and incomplete data are minimal *and* where incomplete data are not extensive. In the event that there are differences between individuals with complete or incomplete observations, population parameters estimated in the analysis (such as means, proportions or regression coefficients) may be biased in ways which are difficult to predict. Further, if missing data are extensive then the precision of estimates may be reduced, often leading to standard errors being inflated, with the implication that a difference may not be declared when in fact it exists (i.e. a type II error).

A number of approaches exist to analyse data where those with complete and incomplete values differ systematically. All standard approaches assume that differences between the missing values and observed values can be related to information collected elsewhere in the dataset (the MAR assumption). Multiple imputation (MI) is a general approach to producing valid inferences when analysing partially observed epidemiological data.[2] The theoretical basis for the approach was

developed by Rubin[1,3] and it has been recommended for use in clinical and epidemiological analyses.[2] MI typically assumes that data are MAR although where theoretically justified it can also assume MNAR.[2] The two potential advantages of MI in our analysis were that MI theoretically produces unbiased population parameters, and is statistically more efficient because all individuals contribute data to the analysis and therefore the statistical power to estimate parameters precisely is retained.

It is important to note that it is impossible to verify why data are missing (i.e. how far the MCAR, MAR or MNAR assumptions are correct) in any given analysis although the data can be used to support whether the MCAR assumption is plausible or not. Therefore, Sterne et al. [2] have developed guidelines for the use and reporting of MI in clinical and epidemiological analysis (Table 1). This supplement is structured with reference to these guidelines to provide: a comprehensive account of how missingness was addressed using MI in our study; why we chose this approach; and analyses of data which support this approach.

Table 1: Guidelines for reporting analysis potentially affected by missing data

**Guideline and notes adapted from Sterne et al [2]**
1. Report the number of missing values for each variable of interest.
2. If possible, give reasons for missing values, in terms of other variables
3. Indicate how many individuals were excluded because of missing data when reporting the flow of participants through the study.
4. Clarify whether there are important differences between individuals with complete and incomplete data
5. Describe the type of analysis used to account for missing data (e.g., MI), and the assumptions that were made (e.g. missing at random)
6. Report details of the software used and of key settings for the imputation modelling
7. Report the number of imputed datasets that were created (five imputed datasets have been suggested to be sufficient on theoretical grounds, but a larger number may be preferable to reduce sampling variability from the imputation process)
8. What variables were included in the imputation procedure?
9. How were non-normally distributed and binary/categorical variables dealt with?
10. If statistical interactions were included in the final analyses, were they also included in imputation models?
11. If a large fraction of the data is imputed, compare observed and imputed values
12. Where possible, provide results from analyses restricted to complete cases, for comparison with results based on MI. If there are important differences between the results, suggest explanations, bearing in mind that analyses of complete cases may suffer more chance variation, and that under the missing at random assumption MI should correct biases that may arise in complete cases analyses.
13. Discuss whether the variables included in the imputation model make the missing at random assumption plausible.
14. It is also desirable to investigate the robustness of key inferences to possible departures from the missing at random assumption, by assuming a range of missing not at random mechanisms in sensitivity analyses. This is an area of ongoing research

S3. Results relating to Sterne et al guidelines

G1. Report the number of missing values for each variable of interest.

Our analyses directly used six variables (the 'variables of interest') from the MEND 7-13 service dataset which had varying proportions of missingness. While other variables (outcome variables) also had missing data, these were included in the model for reasons of good practice (described in more depth in section G8. Table 2 shows what proportion of each variable of interest was missing. It is of note that a relatively large proportion of data was missing for the employment status variable. To decide whether this amount of data could be theoretically imputed we referred to peer-reviewed work, finding work published in the British Medical Journal where the authors have imputed outcomes with 70-75% of observations missing.[4]

Table 2: Distribution of missing data on variables of interest (N=13,998)

Variable	Missing data N	(%)
Ethnic group	4,595	33
Family structure	5,028	36
Housing tenure	4,949	35
Employment status	8,804	63
Attendance	5,811	42
SDQ baseline	1,000	7

G2. If possible, give reasons for missing values, in terms of other variables

It is not possible to know exactly why data were missing, i.e. whether missingness was introduced by MCAR, MAR or MNAR. We hypothesised that data were missing for two broad reasons: missingness introduced by differential reporting by socio-economic and ethnic groups, and missingness introduced by differential amounts of errors at the data collection and data entry stages.

Analyses of the Millennium Cohort Study show that the ethnic background of mothers, the socio-economic status of the ward they live in, family structure, housing tenure and household income are all associated with variations in response. [5,6] These results reflect a wider empirical literature which also reports socio-economic and ethnic variations in response.[7] Hypotheses of why these associations arise are typically broad in nature, reflecting the heterogeneity of ethnic minority and socio-economic groups. For example, Allison et al. [7] suggest that literacy and English comprehension amongst "some sections" of ethnic minority communities might be low and therefore present barriers to responding to surveys designed by white groups from favourable socio-economic circumstances.

All variables with missing data (listed in G8) were collected and entered at local MEND programmes. Therefore, if data were missing because of errors in data collection or data entry, these might be expected to vary with the staff and procedures in place for each programme. We derived variables at the programme level which might be expected to be associated with these variations in missingness between programmes including: the numbers of programmes delivered by each programme manager to date; programme group size; and variables measuring the proportion of height and weight measures which were digit rounded.

Employment status was not collected in 2007 and 2008 and so was not present for those years. Other variables which were observed in these years (ethnic group, family structure and housing tenure) did not vary in their proportions between 2007/08 and 2009/10. On this basis, we assumed that employment status questions for those years could be imputed from values collected in 2009 and 2010.

G3. Indicate how many individuals were excluded because of missing data when reporting the flow of participants through the study.

Overall, MEND collected 21,503 records of families who were referred to the programme and contacted MEND by telephone. 3,214 of these were duplicates (n=371), had incomplete data on age, sex and postcode (n=2,471), or were out of the age range of 6-13 (n=372). Of the 18,289 remaining 'referrals', 13,998 had BMI measured at the first measurement session, and were designated 'starters' on this basis. These 13,998 had incomplete data on the variables of interest and taking a complete case approach would have left a complete case sample of 2,787, excluding 11,211 individuals. These flows are shown in the paper in Figure 1.

G4. Clarify whether there were important differences between individuals with complete and incomplete data

We observed large differences between the distribution of SDQ, housing tenure, and smaller differences in the distribution of adiposity, ethnicity, employment status, attendance, and area deprivation when comparing the proportions of the complete and incomplete data. There were no differences in the distributions of respondents with complete and incomplete data by sex, family structure, or urban/rural status (Table 4).

Therefore, while we expect that analyses based on complete data might underestimate the proportion of abnormally distressed children, and those from private and social renting households.

Table 3: Sensitivity analysis: differences between individuals with complete (N=2,787) and incomplete (N=11,211) data

	Complete		Incomplete		Chi
**Variables**	**%**	**N**	**%**	**N**	**p**
Adiposity					
Overweight	18.7	520	15.0	1,682	<0.001
Obese	81.3	2,267	85.0	9,529	
Baseline SDQ					
Normal	55.9	1,558	49.0	5,488	<0.001
Borderline	14.3	399	13.1	1,464	
Abnormal	29.8	830	38.0	4,259	
Sex					
Boy	54.8	1,528	53.9	6,040	ns
Girl	45.2	1,259	46.1	5,171	
Ethnicity					
White	76.4	2,130	73.9	4,886	
Asian	10.4	291	11.1	732	0.051
Black	7.9	219	9.2	610	
Other	5.3	147	5.9	388	
Family structure					
Lone parent	35.0	976	34.9	2,156	ns
Couple	65.0	1,811	65.1	4,027	
Housing tenure					
Owned	57.9	1,614	50.3	3,148	<0.001
Social	28.0	779	34.7	2,175	
Private	14.1	394	15.0	939	
Employment status					
Employed	74.5	2,076	71.9	1,731	0.037
Unemployed	25.5	711	28.1	676	
Urban/rural					
Urban	88.6	2,470	89.1	9,988	ns
Suburban	6.3	175	6.5	733	
Rural	5.1	142	4.4	490	
Attendance					
Non-completer	8.8	245	11.3	611	
Partial completer	24.9	694	23.9	1,289	0.002
Completer	66.3	1,848	64.8	3,500	
IDACI deciles					
Least deprived	6.6	184	5.9	662	
2	7.6	213	6.7	753	
3	6.8	189	6.2	698	
4	9.1	253	7.6	857	
5	7.2	200	6.8	763	<0.001
6	9.4	262	10.7	1,205	
7	11.9	333	11.7	1,316	
8	14.2	397	12.9	1,444	
9	14.5	405	15.3	1,719	
Most deprived	12.6	351	16.0	1,794	

G5. Describe the type of analysis used to account for missing data (e.g., MI), and the assumptions that were made (e.g. missing at random)

We decided to use MI, making the assumption that data were missing at random (MAR), to account for missing data in the MEND study. Our rationale was based on our hypotheses developed in G2, that missing data were missing due to factors which were observed in the dataset and could be modelled using a multiple imputation model.

G6. Report details of the software used and of key settings for the imputation modelling

We used REALCOM-IMPUTE Software for Multilevel MI with Mixed Response Types. [8] REALCOM-IMPUTE is general use software developed for the applied researcher. As such, the algorithms have been developed and tested for use across a variety of settings. This contrasts with software developed specifically within one context and which may not be applicable in other contexts, a potential software limitation discussed by Sterne et al.[2]

G7. Report the number of imputed datasets that were created

We generated 10 datasets – double the number broadly recommended by Sterne et al. [2] Imputing more datasets reduces sampling variability from the imputation process. We ran 3,000 iterations model, 500 iterations for burn in and a further 2,500, yielding 10 datasets, drawn after 250 iterations. We tested the model for sensitivity to the number of iterations, running earlier models for differing lengths (for example 1000 burn in, drawing datasets at 2,500 iterations). Estimates of analyses did not vary substantively using different numbers of iterations.

G8. What variables were included in the imputation procedure?

REALCOM-IMPUTE was used to impute the parameters listed below. These included the measured variables of which the responses are those with missing data and the ‘predictors’ are those which were completely observed. The parameters also included a random intercept term which varied for each programme and estimated the proportion of missing data at the programme level.

Responses (those with missing data), all of which were individual level variables (level 1 in model)

1. Ethnic group (modelled as unordered categorical)
2. Family structure (unordered categorical)
3. Housing tenure (unordered categorical)
4. Employment status (unordered categorical)
5. SDQ at baseline (continuous)
6. % of sessions attended (continuous)
7. BMI at follow up (continuous)*
8. SDQ at follow up (continuous)*
9. Baseline and follow up self-esteem, diet, physical activity (all continuous)*

Measured predictors (completely observed), individual (level 1) and programme level (level 2)

1. Age and sex (level 1)*
2. Neighbourhood IDACI score (level 1)

3. Urban /rural status (level 1)*
4. Density of unhealthy and healthy food outlets (level 1)*
5. Built environment (density of roads and green space, level 1)*
6. Number of programmes delivered by programme manager (level 2)
7. Size of MEND group at start of programme (level 2)
8. Proportion of height measures rounded to 0 or 0.5cm (level 2)
9. Proportion of weight measures in programme rounded to 0 or 0.5kg (level 2)

Variables which were starred in the lists above were included in the MI model but were not theorised to be associated with missingness on the variables of interest here (i.e. ethnicity, family structure, housing tenure, employment status, attendance, or baseline SDQ). These variables were included because they were used in other analyses of the data reported elsewhere as part of our wider study of the MEND data and good imputation practice suggests that all variables to be included in any later models of interest are included in imputation models to ensure that relationships between variables are not under-estimated. [2]

G9. How were non-normally distributed and binary/categorical variables dealt with?

REALCOM-IMPUTE was developed specifically to robustly handle mixed response types and the statistical theory and equations are described by Goldstein et al. [9] while the software itself and the way that it implements these is described by Carpenter et al. [8] We followed these procedures.

G10. If statistical interactions were included in the final analyses, were they also included in imputation models?

No statistical interactions were included in the final analyses.

G11. If a large fraction of the data is imputed, compare observed and imputed values

There is no consensus to our knowledge about what constitutes 'too high' a fraction of missing data.[10] As described above, the amount missing on any given variable ranged from 7 to 63%. While 7% is possibly not a 'large fraction' we compare the observed values against the imputed values for all the variables for completeness in Table 4.

Proportions on all variables are identical for baseline SDQ. Proportions but not confidence intervals were identical or very similar for family structure, housing tenure and employment status although confidence intervals differed slightly. Proportions were similar for ethnicity with white and Asian families estimated to be slightly higher in imputed data, and black and other families estimated to be slightly lower in imputed data. Imputed and observed values differed most for attendance: completion and non-completion was estimated to be lower in imputed data while partial completion was estimated to be higher.

Overall, given the large proportion of missing on variables such as employment status, there are few large differences between the observed and imputed values. Only completion differs slightly, and the difference is as might be expected. Analyses for guideline 4 showed that lower socio-economic groups were under-represented in those with complete case data, and less favourable socio-economic circumstances have been associated with higher attrition in paediatric weight management interventions. [11] Thus, it might be expected that completion would be over-estimated using only observed values.

Table 4: Proportions calculated from observed values (using casewise deletion so N varies) and imputed values (N=13,998)

	Observed (N varies)		Imputed (N=13,998)	
**Variables**	**Prop. (95%CI)**	**N**	**Prop. (95%CI)**	**Percentage imputed**
SDQ baseline				
Normal	0.54 (53, 55)	12,998	0.54 (0.53,0.55)	7%
Borderline	0.14 (0.14,0.15)		0.14 (0.14,0.15)	
Abnormal	0.31 (0.31, 0.32)		0.31 (0.31,0.32)	
Ethnicity				
White	0.75 (0.74,0.75)		0.77 (0.76,0.78)	
Asian	0.11 (0.1,0.12)	9,403	0.13 (0.12,0.14)	33%
Black	0.09 (0.08,0.09)		0.06 (0.06,0.06)	
Other	0.06 (0.05,0.06)		0.04 (0.04,0.04)	
Family structure				
Lone parent	0.35 (0.34,0.36)	8,970	0.35 (0.34,0.35)	36%
Couple	0.65 (0.64,0.66)		0.65 (0.65,0.66)	
Housing tenure				
Owned	0.53 (0.52,0.54)	9,049	0.53 (0.52,0.55)	35%
Social	0.33 (0.32,0.34)		0.32 (0.31,0.33)	
Private	0.15 (0.14,0.15)		0.15 (0.14,0.16)	
Employment status				
Employed	0.73 (0.72,0.74)	5,194	0.74 (0.72,0.76)	63%
Unemployed	0.27 (0.26,0.28)		0.26 (0.24,0.28)	
Attendance				
Non-completer	0.10 (0.1,0.11)	8,187	0.08 (0.07,0.08)	42%
Partial completer	0.24 (0.23,0.25)		0.33 (0.32,0.34)	
Completer	0.65 (0.64,0.66)		0.60 (0.58,0.61)	

G12. Provide results from analyses restricted to complete cases, for comparison with results based on MI.

In the sensitivity analyses below we present data for the imputed datasets (as in the paper) and for complete case (CC) samples where data is complete for all the variables of interest. Given the high missing data on parental employment, we also explore the influence of this variable on the results, by generating a 'complete case' dataset which was complete for all variables except employment status (equivalent to excluding employment status from the analysis).

Table 5 and Table 6 show differences in proportions calculated for imputed and both complete case datasets for starters (Table 5) and completers (Table 6) respectively. The results show that the conclusion we draw in the paper - that "the provision and / or uptake of MEND did not appear to compromise, and if anything, promoted participation among those from more disadvantaged circumstances and from ethnic minority groups" – was consistent across imputed and complete case datasets.

Table 7 shows the relative risks of completion for imputed data and the two types of complete case analyses. The relative risks do not vary in direction or magnitude. This shows that the conclusion in the paper, that – "completion was relatively less likely for those participants living in less favourable socio-economic circumstances" - was consistent across imputed and complete case datasets.

Table 5: Differences in proportions between MEND-eligible and MEND starters: using imputed (N=13,998), complete case (CC, N=2,787), and CC excluding employment status (N=5,191) data

	Imputed		Complete case		Complete case excluding employment	
**Variables**	**Diff.**	**(p values)**	**Diff.**	**(p values)**	**Diff.**	**(p values)**
Adiposity						
Overweight excl. obese	-38.1	(<0.001)	-35.1	(<0.001)	-37.9	(<0.001)
Obese	+38.1	(<0.001)	+35.1	(<0.001)	+37.9	(<0.001)
Sex						
Boy	-7.1	(<0.001)	-7.8	(<0.001)	-8.0	(<0.001)
Girl	+7.1	(<0.001)	+7.8	(<0.001)	8.0	(<0.001)
Ethnicity						
White	-2.3	(0.01)	-3.2	(0.004)	-3.1	(0.001)
Asian	+3.0	(<0.001)	+0.4	(0.579)	-0.1	(0.833)
Black	+0.2	(0.6)	+2.2	(0.001)	+2.5	(<0.001)
Other	-0.9	(<0.03)	+0.5	(0.348)	+0.8	(0.144)
Family structure						
Lone parent	+4.0	(0.02)	+4.5	(0.018)	+4.1	(0.022)
Couple	-4.0	(0.02)	-4.5	(0.018)	-4.1	(0.022)
Housing tenure						
Owned	-10.1	(<0.001)	-5.6	(<0.001)	-8.6	(<0.001)
Social	+6.7	(<0.001)	+2.8	(0.019)	+5.9	(<0.001)
Private	+3.4	(<0.001)	+2.8	(0.002)	+2.7	(0.001)
Employment status						
Employed	-5.5	(<0.001)	-4.9	(<0.001)	Excluded	
Unemployed	+5.5	(<0.001)	+4.9	(<0.001)		
Urban/rural						
Urban	+6.9	(<0.001)	+6.6	(<0.001)	+6.8	(<0.001)
Suburban	-2.5	(<0.001)	-2.5	(<0.001)	-2.7	(<0.001)
Rural	-4.5	(<0.001)	-4.5	(<0.001)	-4.0	(<0.001)

Table 6: Differences in proportions between MEND-eligible and MEND completers: using imputed (N=8,311), complete case (CC, N=1,848), and CC excluding employment status (N=3,465) data

	Imputed		Complete case		Complete case excluding employment	
Variables	Diff.	(p values)	Diff.	(p values)	Diff.	(p values)
Adiposity						
Overweight excl. obese	-38.0	(<0.001)	-35.0	(<0.001)	-38.1	(<0.001)
Obese	+38.0	(<0.001)	+35.0	(<0.001)	+38.1	(<0.001)
Sex						
Boy	-9.6	(<0.001)	-10.8	(<0.001)	-10.5	(<0.001)
Girl	+9.6	(<0.001)	+10.8	(<0.001)	+10.5	(<0.001)
Ethnicity						
White	-1.1	(0.3)	-4.3	(0.2)	-1.8	(0.089)
Asian	+2.4	(<0.003)	-0.6	(0.8)	-0.6	(0.461)
Black	-0.2	(0.6)	+1.6	(0.03)	+1.9	(0.003)
Other	-1.1	(0.02)	+0.2	(0.8)	+0.4	(0.458)
Family structure						
Lone parent	+1.0	(0.6)	+1.0	(0.6)	+0.4	(0.824)
Couple	-1.0	(0.6)	-1.0	(0.6)	-0.4	(0.824)
Housing tenure						
Owned	-5.3	(<0.001)	0.0	(>0.9)	-3.1	(0.011)
Social	+2.7	(0.009)	-1.8	(0.2)	+1.4	(0.215)
Private	+2.6	(0.001)	+1.9	(<0.06)	+1.7	(0.037)
Employment status						
Employed	-2.2	(0.04)	-0.8	(0.6)	Excluded	
Unemployed	+2.2	(0.04)	+0.8	(0.6)		
Urban/rural						
Urban	+6.0	(<0.001)	+6.3	(<0.001)	+6.1	(<0.001)
Suburban	-1.9	(0.001)	-2.5	(0.003)	-2.4	(<0.001)
Rural	-4.1	(<0.001)	-3.8	(<0.001)	-3.7	(<0.001)

Table 7: Multivariable model of completion: imputed (N=13,998), complete case (CC, N=2,787), and CC excluding employment status (N=5,191)

	Imputed	Complete case	Complete case excluding. employment
Parameters	aRR (95% CI)	aRR (95% CI)	aRR (95% CI)
Intercept	0.85 [0.76,0.95] **	0.98 (0.78, 1.24)	
SDQ baseline (ref. 'Normal')			
'Borderline'	0.97 [0.90,1.04]	0.98 [0.86,1.13]	1.00 [0.91,1.11]
'Abnormal'	0.91 [0.86,0.97] **	0.93 [0.83,1.03]	0.93 [0.86,1.00]
Sex (ref. Girls)			
Boys	0.91 [0.87,0.96] ***	0.89 [0.81,0.98] *	0.90 [0.85,0.97] **
Family structure (ref. couple)			
Lone parent	0.93 [0.88,0.98] *	0.94 [0.85,1.05]	0.91 [0.84,0.98] *
Housing tenure (ref. Owner			
Social rented	0.88 [0.82,0.95] ***	0.85 [0.74,0.97] *	0.85 [0.78,0.93] ***
Private rented	0.90 [0.84,0.97] **	0.91 [0.78,1.05]	0.89 [0.80,0.99] *
Employed status (ref. Employed)			
Unemployed	0.93 [0.87,0.98] *	0.91 [0.80,1.04]	Excluded.
IDACI 2007 deciles (ref. Decile 1,			
2	1.01 [0.89,1.15]	1.03 [0.82,1.29]	0.97 [0.82,1.15]
3	0.99 [0.88,1.12]	1.04 [0.82,1.31]	0.99 [0.83,1.18]
4	0.98 [0.87,1.11]	0.91 [0.72,1.14]	0.91 [0.77,1.08]
5	0.96 [0.84,1.08]	0.98 [0.78,1.24]	0.97 [0.82,1.16]
6	0.92 [0.82,1.03]	0.96 [0.77,1.20]	0.93 [0.79,1.09]
7	0.93 [0.82,1.04]	0.92 [0.74,1.15]	0.88 [0.75,1.04]
8	0.93 [0.82,1.05]	0.98 [0.79,1.21]	0.95 [0.81,1.11]
9	0.91 [0.80,1.02]	0.88 [0.71,1.09]	0.89 [0.76,1.04]
10 - most deprived	0.85 [0.76,0.96] **	0.89 [0.71,1.12]	0.83 [0.71,0.98] *
Programme group size (ref. 1-5)			
6-9	0.93 [0.86,1.01]	0.88 [0.74,1.03]	0.90 [0.79,1.02]
10 or more	0.84 [0.77,0.91] ***	0.80 [0.67,0.95] *	0.82 [0.72,0.94] **
Number of programmes per			
10 or more	0.93 [0.86,0.99] *	0.95 [0.86,1.05]	0.91 [0.84,1.00] *

*p<0.05, **p<0.01, ***p<0.001

G13. Discuss whether the variables included in the imputation model make the missing at random assumption plausible.

Referring back to the discussion of reasons for missingness in guideline 2, we hypothesised that missingness would be associated with between-group differences on ethnic and socio-economic variables. We also discussed how missingness on all variables would be expected to vary systematically between programmes and that variables measured at the programme level relating to data quality, staff experience and group size might be expected to be associated with missingness.

Therefore, the plausibility of the MAR assumption would be supported by evidence showing that missingness on the variables of interest varied systematically between programmes and that missingness was associated with proxies for the variables mentioned. In the following analysis we aim to test whether these associations support the imputation model described above.

We assessed whether the missing at random possibilities by constructing six binary variables marking where each of the variables of interest above was missing (coded 1) or observed (coded 0). We used multilevel poisson regression models to model each missingness outcome with no covariates (i.e. six variance components model). We then used Equation 1 to calculate the proportion of variation in missing data on each variable that was attributable to systematic differences between programmes.

Equation 1: 'Exact calculation method' of estimating variance partition coefficient (VPC) in multilevel poisson regression models [12]

$$VPC = \frac{[exp(2X\beta + 2\sigma^2) - exp(2X\beta + \sigma^2)]}{\left[exp(2X\beta + 2\sigma^2) - exp(2X\beta + \sigma^2) + exp\left(2X\beta + \frac{\sigma^2}{2}\right)\right]}$$

Where VPC is the variance partition coefficient, $X\beta$ is the fixed part of the model (in the case of these variance components models this is the intercept term and is a constant) and σ^2 which is the level 2 variance term.

96% of missingness on attendance was explained by systematic variation between programmes. This is plausible because families have no role in the data collection process; missing data arise on this variable because it is entered erroneously or not at all by programme staff. However, high proportions of the missingness on other variables were also explained by the programme level, ranging from 25% to 69% - again consistent with the reasons outlined in guideline 2 above, that surveys were collected and entered by programme staff. Unfortunately, the model for baseline SDQ did not converge and so the proportion of missing data attributable to programme level variation could not be estimated.

Table 8: Multilevel variance components models of variables marking missing data where level 1 is participants in MEND and level 2 is MEND programmes (N=13,998, N programmes = 1,940)

Missingness	$X\beta$	σ^2	VPC
Ethnic group	-1.78	1.83	0.69
Family structure	-1.48	1.16	0.47
Housing tenure	-1.57	1.36	0.55
Employment status	-0.64	0.41	0.25
Attendance	-1.87	3.47	0.96
Baseline SDQ	Model did not converge after 500 iterations		

The second reason for missingness varying was because ethnic minority groups and families from different socio-economic circumstances might vary in their reporting on survey questions. We used poisson regression models (with adjustment for the clustering at programme level so that associations were independent of the clustering of missing data demonstrated above) to estimate whether missingness was associated with other variables in the dataset.

Table 9 shows that missingness on the ethnic and socio-economic variables was associated with observed values on the other socio-demographic variables, neighbourhood deprivation, programme group size and the number of programmes delivered by the programme manager. Missingness on attendance was also associated with group size, the percentage of height measures in the programme which were rounded and with social and private renting.

The data do not support the assumption that missingness was MCAR (i.e. that missingness arose by chance and was not associated with any observed variables).

Overall, the presence of statistically significant associations between missingness on analysis variables supported the rationale developed in guideline 2, that missingness could be explained by observed variables at the family, neighbourhood and programme level.

Table 9: Unadjusted relative risks for variables with missing data and variables in MI model

	Analysis variables with missing data				
	**Ethnic group**	**Housing tenure**	**Family structure**	**Employment status**	**Attendance**
**Variable**	**uRR (95%CI)***	**uRR (95%CI)***	**uRR (95%CI)***	**uRR (95%CI)***	**uRR (95%CI)***
Housing tenure (ref owner occupied)					
Social rented	**1.13 [0.73,1.75]**	-	**1.11 [0.90,1.36]**	**1.19 [1.11,1.27]**	**1.12 [1.03,1.22]**
Private rented	**1.42 [0.86,2.33]**	-	**1.30 [1.01,1.67]**	1.06 [0.98,1.15]	**1.12 [1.02,1.22]**
Ethnic group (ref white)					
Asian	-	1.35 [0.97,1.89]	**2.06 [1.59,2.66]**	0.95 [0.85,1.08]	1.03 [0.88,1.21]
Black	**-**	**1.42 [1.02,1.98]**	**1.56 [1.16,2.10]**	1.07 [0.94,1.22]	0.96 [0.80,1.15]
Other	**-**	1.37 [0.94,1.99]	**1.73 [1.27,2.35]**	1.02 [0.91,1.15]	0.98 [0.84,1.15]
Family structure (ref couple)					
Lone parent	**0.65 [0.46,0.94]**	1.08 [0.89,1.31]		0.97 [0.92,1.02]	1.04 [0.98,1.11]
Employment status (ref employed)					
Unemployed	1.09 [0.64,1.85]	**1.64 [1.16,2.32]**	**1.20 [0.98,1.48]**		1.07 [0.94,1.21]
IDACI 2007	**0.57 [0.46,0.71]**	**0.60 [0.49,0.73]**	**0.66 [0.55,0.80]**	1.01 [0.90,1.15]	1.18 [0.96,1.45]
Built environment	**0.94 [0.90,0.98]**	**0.94 [0.91,0.98]**	**0.94 [0.90,0.98]**	1.01 [0.98,1.03]	0.99 [0.95,1.03]
Urban/rural status (ref urban)					
Suburban	1.05 [0.91,1.20]	1.07 [0.94,1.22]	1.05 [0.93,1.19]	1.02 [0.94,1.10]	0.99 [0.87,1.13]
Rural	1.02 [0.88,1.18]	1.00 [0.87,1.15]	0.99 [0.86,1.14]	0.96 [0.88,1.05]	0.94 [0.81,1.08]
Programme group size	**0.95 [0.93,0.97]**	**0.96 [0.94,0.98]**	**0.96 [0.95,0.98]**	0.99 [0.98,1.00]	**0.96 [0.94,0.98]**
Number of programmes per manager	**0.96 [0.94,0.97]**	**0.96 [0.95,0.97]**	**0.97 [0.96,0.98]**	**0.96 [0.95,0.97]**	0.99 [0.98,1.00]
Percent height rounded	1.01 [1.01,1.01]	1.01 [1.01,1.01]	1.01 [1.00,1.01]	1.01 [1.01,1.02]	**1.02 [1.02,1.03]**
Percent sessions attended	1.00 [1.00,1.00]	1.00 [1.00,1.00]	1.00 [1.00,1.00]	1.00 [1.00,1.00]	-
Percent weight rounded	1.00 [1.00,1.01]	1.00 [1.00,1.00]	1.00 [1.00,1.00]	1.00 [1.00,1.00]	1.00 [1.00,1.01]

*Relative risks calculated using modified Poisson regression; standard errors are adjusted for clustering at the programme level.

G14. Investigate the robustness of key inferences to possible departures from the missing at random assumption, by assuming a range of missing not at random mechanisms in sensitivity analyses.

We could not formulate hypotheses where missing values for our variables might themselves be associated with missingness (i.e. where missingness might be MNAR). In the absence of these hypotheses under which to introduce MNAR assumptions into the MI model for sensitivity analyses, we could not investigate this possibility.

S4. Summary

Missing data were extensive in the MEND service data, which means that MI was more efficient than complete case analysis and would therefore be likely to estimate more precise parameter estimates. Further, we showed that there were statistically significant but small differences between individuals with complete and incomplete data for all variables of interest in the analysis: namely psychological distress, ethnic group, socio-economic circumstances, and attendance. Proportions of missing data were also systematically missing between MEND programmes. This suggested that missing data were not MCAR and that multilevel MI was more likely to produce unbiased estimates of population parameters than complete case analysis.

Our comparison of complete case analyses and imputed analyses showed that there were no substantive differences in findings between the MI and complete case results beyond expected losses of precision related to the large reduction in power in complete case analyses.

Overall, we were confident that our multiple imputation findings were robust and that they were likely to lead us to draw the most valid statistical inferences from the MEND data.

References

1 Rubin DB. Multiple Imputation for Nonresponse in Surveys. New York: John Wiley and Sons; 1987.
2 Sterne JAC, White IR, Carlin JB, Spratt M, Royston P, Kenward MG, et al. Multiple imputation for missing data in epidemiological and clinical research: potential and pitfalls. *BMJ*. 2009;338.
3 Rubin DB. Multiple Imputation After 18+ Years. *J Am Stat Assoc*. 1996;91(434):473-89.
4 Hippisley-Cox J, Coupland C, Vinogradova Y, Robson J, Brindle P. QRISK cardiovascular disease risk prediction algorithm—comparison of the revised and the original analyses: Technical supplement 1. 2007.
5 Dex S, Rosenberg R. Working Paper 2008/11: Ethnic minorities and non-response in the Millennium Cohort Study. London: Centre for Longitudinal Studies, Institute of Education, University of London; 2008.
6 Plewis I. Millennium Cohort Study First Survey: Technical Report on Sampling. London: Centre for Longitudinal Studies, Institute of Education, University of London; 2004.
7 Allison T, Ahmad T, Brammah T, Symmons D, Urwin M. Can Findings from Postal Questionnaires be Combined with Interview Results to Improve the Response Rate among Ethnic Minority Populations? *Ethnicity & Health*. 2003;8(1):63-9.
8 Carpenter JR, Goldstein H, Kenwood MG. REALCOM-IMPUTE Software for Multilevel Multiple Imputation with Mixed Response Types. *J Stat Softw*. 2011;45(5):1-14.
9 Goldstein H, Carpenter J, Kenward M G , Levin K. Multilevel Models with Multivariate Mixed Response Types. *Statistical Modelling*. 2009;9(173):197.
10 Royston P. Multiple imputation of missing values: update. *The Stata Journal*. 2005;5(2):1-14.

11 Skelton JA, Beech BM. Attrition in paediatric weight management: a review of the literature and new directions. *Obesity Reviews*. 2011;12(5):e273-e81.
12 Stryhn H, Sanchez J, Morley P, Booker C, Dohoo IR, editors. Interpretation of variance parameters in multilevel Poisson regression models. 11th International Symposium on Veterinary Epidemiology and Economics; Cairns, Australia; 2006.
